# Cell‐Based Therapies for Degenerative Musculoskeletal Diseases

**DOI:** 10.1002/advs.202207050

**Published:** 2023-05-18

**Authors:** Pengbin Yin, Yuheng Jiang, Xuan Fang, Daofeng Wang, Yi Li, Ming Chen, Hao Deng, Peifu Tang, Licheng Zhang

**Affiliations:** ^1^ Department of Orthopedics the Fourth Medical Center Chinese PLA General Hospital Beijing 100853 China; ^2^ National Clinical Research Center for Orthopedics Sports Medicine & Rehabilitation Beijing 100853 China; ^3^ Department of Orthopedics General Hospital of Southern Theater Command of PLA No. 111, Liuhua Avenue Guangzhou 510010 China; ^4^ Department of Anatomy, Histology and Embryology School of Basic Medical Sciences Peking University Health Science Center Beijing 100191 China; ^5^ Department of Orthopedics Third Affiliated Hospital of Jinzhou Medical University Jinzhou Liaoning Province 121000 China

**Keywords:** cell‐based therapies, degenerative musculoskeletal diseases, exosomes, extracellular vesicles, stem cells

## Abstract

Degenerative musculoskeletal diseases (DMDs), including osteoporosis, osteoarthritis, degenerative disc disease, and sarcopenia, present major challenges in the aging population. Patients with DMDs present with pain, functional decline, and reduced exercise tolerance, which result in long‐term or permanent deficits in their ability to perform daily activities. Current strategies for dealing with this cluster of diseases focus on relieving pain, but they have a limited capacity to repair function or regenerate tissue. Cell‐based therapies have attracted considerable attention in recent years owing to their unique mechanisms of action and remarkable effects on regeneration. In this review, current experimental attempts to use cell‐based therapies for DMDs are highlighted, and the modes of action of different cell types and their derivatives, such as exosomes, are generalized. In addition, the latest findings from state‐of‐the‐art clinical trials are reviewed, approaches to improve the efficiency of cell‐based therapies are summarized, and unresolved questions and potential future research directions for the translation of cell‐based therapies are identified.

## Graphical abstract

Degenerative musculoskeletal diseases (DMDs) pose a serious challenge to the elderly. In this review, the latest results from state‐of‐the‐art basic and clinical research are summarized to illustrate the great potential of cell‐based therapies in the treatment of DMDs. Furthermore, this work highlights the unresolved questions for the translation of cell‐based therapies and points out future research directions.

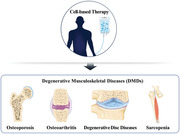

## 1 Introduction

Degenerative musculoskeletal diseases (DMDs) include various age‐related disorders of the musculoskeletal system, among which osteoporosis, osteoarthritis (OA), sarcopenia, and degenerative joint diseases have the highest incidence. Patients with these diseases experience increased pain, a decreased range of motion, and functional deficits.^[^ ^1^, ^2^, ^3^ ^]^ DMDs significantly limit mobility and dexterity and are associated with an increased risk of falling, leading to early retirement from work, lower levels of wellbeing, and a reduced ability to participate in society, all of which can cause a significant reduction in quality of life.^[^ ^4^ ^]^ Degenerative diseases of the musculoskeletal system not only cause serious physical and mental suffering but also increase healthcare costs and the economic burden on the families of patients and healthcare systems.^[^ ^5^ ^]^ According to the *World Population Aging 2020 Highlights*, there will be 727 million people aged 65 years or older globally by 2020.^[^ ^6^ ^]^ With the aging of the population, the prevalence of musculoskeletal conditions has surged and is expected to continue increasing over the next decade. This will cause an increase in serious social and health problems.^[^ ^7^ ^]^ Developing effective treatments for DMDs is, therefore, imperative and needs to be addressed.

There are two main categories of in‐service clinical treatment strategies for DMDs: conservative therapy and surgical treatment. Surgery is commonly considered the last treatment option for DMDs. Previously, painkillers were the most commonly used medications for DMDs, including for OA and degenerative disc diseases (DDDs). However, this symptom‐oriented approach cannot prevent the progression of degeneration. Therefore, patients inevitably undergo surgical interventions. Various drugs, such as bisphosphonates, can slow the progression of osteoporosis. However, serious complications, such as atypical subtrochanteric fractures and osteonecrosis of the jaw, are associated with additional risks. Therefore, there is an urgent need to develop tissue regeneration approaches to stop the degenerative process, repair defective tissues, and restore musculoskeletal functions.

**Figure 1:**
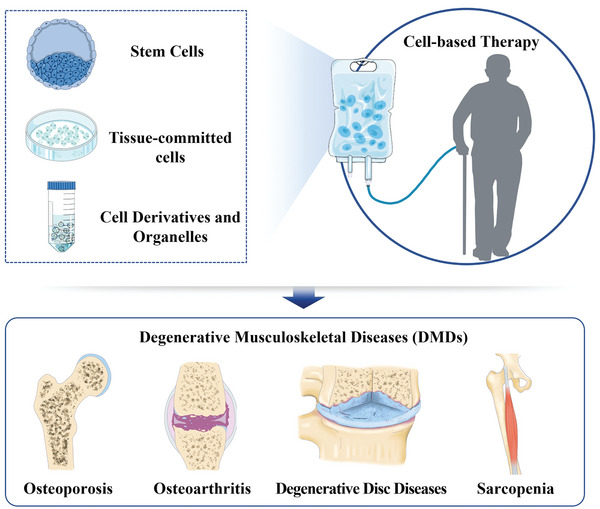
Different types of cells and their derivatives are used in the treatment of DMDs.

Cell‐based therapies have great potential in the field of regenerative medicine. Different cell types and their derivatives have been used to treat diseases other than DMDs, such as cardiovascular disease, cancer, and neurological disorders.^[^ ^8^, ^9^, ^10^ ^]^ Several comprehensive reviews have summarized recent advances in cell‐based therapies.^[^ ^11^, ^12^ ^]^ Many attempts have been made to use cell‐based therapies to treat DMDs and have produced promising results. Some of these regenerative strategies have shown encouraging results in preclinical studies and have been demonstrated to be safe in clinical trials. However, many key problems remain to be solved, such as safety concerns and a lack of standardized processes. In this review, an overview of previous attempts to use cell‐based therapies is provided and the status of cell‐based therapies aimed at counteracting musculoskeletal conditions is described. The latest findings in the field are further summarized, major unresolved questions are identified, current challenges are presented, and recommendations for future research and clinical trials are provided (**Figure** **1**).

## 2 Current Clinical Therapies for DMDs

### 2.1 OA

OA is the most common cause of lower‐extremity pain in elderly people. Patients with OA experience pain, stiffness, and functional impairment of the damaged joints. Analgesia is the most important treatment for OA. Painkillers and surgery are often used to reduce joint pain. The use of painkillers, such as nonsteroidal anti‐inflammatory drugs (NSAIDs), is typically accompanied by complications, such as peptic ulcers, various types of renal insufficiency, and an increased risk of adverse cardiovascular events. A study involving >80 000 patients with OA showed a significant increase in all‐cause mortality over a 1‐year follow‐up period assessing the use of painkillers, such as tramadol.^[^ ^13^ ^]^ End‐stage surgical treatments, such as arthrodesis and joint replacement, are capable of relieving pain but are deficient in reversing disease progression because they aim to achieve symptom remission but do not resolve the underlying cause.^[^ ^14^ ^]^ In addition, there are currently no interventions that restore damaged or degraded articular cartilage.

### 2.2 Sarcopenia

Sarcopenia is a progressive skeletal muscle disorder involving an accelerated loss of muscle mass and function. Adverse outcomes, such as falls, frailty, functional impairment, and mortality, tend to increase as the disease progresses. The benefits of pharmacological interventions in the treatment of sarcopenia remain unclear. Several trials have used selective androgen receptor modulators or myostatin antibodies to treat patients with sarcopenia. However, their clinical efficacy remains unclear. Given the absence of effective drugs, physical activity, and nutritional interventions are currently recommended for patients.^[^ ^15^ ^]^ These treatment options depend on the patient's physical condition, meaning that they are ineffective for people with weaker physical conditions. Therefore, there is an urgent need to develop new drugs that can restore muscle mass and function.

### 2.3 DDDs

DDDs, also referred to as intervertebral disc (IVD) degeneration, comprise a class of age‐related IVD disorders and are a common cause of chronic lower back pain (LBP). Nonsurgical treatment usually involves the use of NSAIDs to relieve pain. Benzodiazepines can also be used to relax muscles; however, they have sedative side effects that limit the ability of patients to work and drive. Patients with severe symptoms that are not relieved by medication require surgery, typically spinal fusion or disc replacement. However, after spinal fusion, patients lose spinal mobility, their spinal biomechanics are altered, and there is an increase in long‐term degenerative changes in the adjacent vertebral segments. Disc replacement theoretically preserves the normal range of spinal motion; however, several studies have shown that there is no clinical difference between the two types of surgeries in relieving LBP.^[^ ^16^ ^]^ Given the side effects of current drug treatments and the lack of surgical effectiveness, new therapies aimed at terminating the progression of degeneration are urgently needed.^[^ ^17^ ^]^

### 2.4 Osteoporosis

Osteoporosis is the most common degenerative disease of the musculoskeletal system, and bisphosphonates are first‐in‐class clinical treatments. Bisphosphonates inhibit bone resorption and slow the progression of osteoporosis. Studies have shown that bisphosphonates can increase bone mineral density in men and postmenopausal women with osteoporosis.^[^ ^18^ ^]^ A systematic review of trials published between 2005 and 2019 demonstrated that bisphosphonates reduced the occurrence of fragility fractures caused by osteoporosis.^[^ ^19^ ^]^ Other drugs, such as estrogens, selective estrogen receptor modulators, denosumab, and teriparatide, have also been shown to have effective therapeutic effects. However, the currently used therapies do not meet the needs of all patients and have considerable side effects that compromise their long‐term use.^[^ ^20^, ^21^ ^]^ For example, osteonecrosis of the jaw and atypical subtrochanteric and diaphyseal femoral fractures are serious complications associated with BP use. A prospective cohort study of 196 129 women aged ≥50 years who were using bisphosphonates found that 277 atypical femoral fractures occurred during the 10 years of follow‐up.^[^ ^22^ ^]^ These complications seriously affected the quality of life of the patients and the efficacy of treatment.

In general, there are two main categories of clinical treatment options for DMDs: those that relieve symptoms, such as painkillers for OA and DDD, and those that slow disease progression, such as antiresorptive drug therapy for osteoporosis, and exercise and nutritional intervention for sarcopenia. Although different approaches have been used to treat musculoskeletal degenerative diseases, existing clinical treatments are not perfect. Current treatments cannot solve the problem of overall insufficient regeneration due to degenerative diseases of the musculoskeletal system at the overall level, therefore, cannot decelerate or reverse disease progression. Cell therapy is the most promising candidate for treating such diseases.

## 3 Overview of Cell Types and Mechanisms of Cell‐Based Therapy

In 1931, Paul Niehans injected the parathyroid glands of steers to treat a patient with erroneous parathyroid gland removal. This is believed to be the first attempt to use cell‐based therapy.^[^ ^23^ ^]^ Since then, bone marrow transplantation has emerged as the most common and well‐established cell transplantation therapy and has been the only clinically applicable therapy for a long time. Bone marrow‐derived cells are injected into patients with conditions such as acute myeloid leukemia and home into the afflicted area to reestablish biological functions, such as hematopoiesis.^[^ ^24^ ^]^ Cell‐based therapy involves the injection, grafting, or transplantation of viable cells or cellular materials into a patient to exert therapeutic effects.

**Figure 2:**
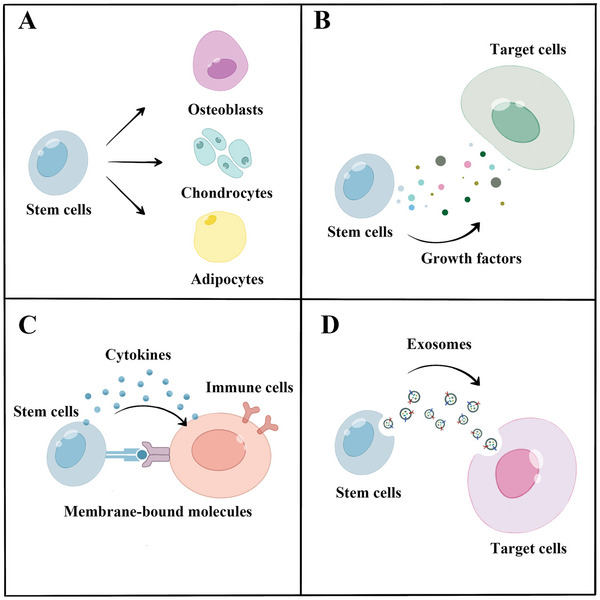
Mechanisms of cell‐based therapy. A) Stem cells differentiate or replace specific cells. B) Stem cells produce various growth factors that exert trophic functions. C) Stem cells play an immunomodulatory role by exerting biological effects on various types of immune cells through cytokines in a non‐contact manner or through membrane‐bound molecules in a contact manner. D) Exosomes from stem cells play a regulatory role as mediators of intercellular communication.

Over the past few decades, various cell types and their derivatives have been studied for use in cell‐based therapies. Different cell types exhibit varying capacities and activities for cell‐based therapies. Stem and tissue‐committed cells are the two primary cell types used for cell therapy. Mesenchymal stem cells (MSCs), induced pluripotent stem cells (PSCs) (iPSCs), embryonic stem cells (ESCs), and muscle satellite cells (SCs) are among the stem cells that are frequently employed in cell therapy for musculoskeletal system disorders, whereas chondrocytes are the most frequently used tissue‐committed cells. Additionally, the use of cell derivatives, such as exosomes, has significantly increased in recent years, increasing their potential for cell therapy. In this section, several common cell types used in cell‐based therapies for DMDs are introduced. The definitions, origins, functions, and mechanisms of various cell types and their derivatives are discussed (**Figure** **2**).

### 3.1 Tissue‐Committed Cells

The use of tissue‐committed cells as a form of cellular therapy to treat degenerative diseases of the musculoskeletal system has been explored. Chondrocytes and IVD cells are the most common cell types. Recently, direct chondrocyte transplantation has been used to treat patients with OA in clinical settings. IVD cell transplantation is also used to treat DDD. Biological functions are mostly achieved through the transplantation of tissue‐committed cells, in which senescent cells are directly replaced by new cells. Section 4 of this review elaborates on this aspect.

### 3.2 Stem Cells

#### 3.2.1 MSCs

MSCs, also known as mesenchymal stromal cells, are among the most investigated cell types for cell‐based therapies. MSCs can be isolated from many different tissues, such as bone marrow, peripheral blood, adipose tissue, and umbilical cords. Studies have shown that MSCs exhibit low immunogenicity and can suppress immune responses via immunomodulatory factors.^[^ ^25^ ^]^ Meirelles et al. summarized the immunomodulatory factors secreted by MSCs, including PGE‐2, TGF‐*β*, HGF, IDO, and iNOS. These factors help MSCs interact with various immune cells to exert immunoregulatory effects. MSCs have been shown to directly suppress *αβ* and *λδ* T cells, inhibit B‐cell proliferation and natural killer (NK) cell activation, and affect the function of dendritic cells and macrophages.^[^ ^26^ ^]^

Numerous studies have demonstrated the ability of MSCs to differentiate directly. MSCs from humans, mice, rats, rabbits, and dogs can differentiate into terminally differentiated mesenchymal cells, such as osteoblasts (bone cells), chondrocytes (cartilage cells), myocytes (muscle cells), and adipocytes (fat cells), both in vivo and in vitro.^[^ ^27^ ^]^ This differentiation potential makes MSCs an ideal source of cells for tissue regenerative medicine. For example, He et al. used a hydroxyapatite‐collagen matrix to induce MSC differentiation into an osteoblastic lineage and successfully observed intramembranous osteogenesis.^[^ ^28^ ^]^ Chen et al. combined collagen hydrogels and chondrogenesis‐inducing factors to successfully induce MSCs differentiation into chondrocytes.^[^ ^29^ ^]^ Tu et al. used aligned electrospun fibers modified with a soluble tendon‐derived extracellular matrix (ECM) as a scaffold to culture MSCs and observed tenogenic differentiation in vitro and rat Achilles tendon regeneration in vivo.^[^ ^30^ ^]^ Thus, the differentiation potential of MSCs significantly broadens their application in cell‐based therapies.

Despite their differentiation potential, MSCs primarily promote tissue repair and regeneration through paracrine activity. MSCs secrete soluble molecules that act on target organs and cells. These molecules include growth factors, cytokines, and hormones. MSCs exert their trophic functions through cytokines, thereby activating or recruiting other progenitor cells to the site of injury to perform their biological functions. For example, MSCs recruit neighboring endothelial progenitor cells by producing Ang‐1 and CXCL12.^[^ ^31^ ^]^ A recent study also found that MSCs secrete CXCL14 and CCL2, which are beneficial for recruiting C‐X‐C motif receptor 4+ (CXCR4+) and chemokine receptor‐2+ vessel‐associated cells.^[^ ^32^ ^]^ Given their potential for differentiation, ease of availability, and low immunogenicity, MSCs are among the most attractive cell types for cell‐based therapies.

#### 3.2.2 ESCs

ESCs, which may be derived from the inner cell mass of an embryo that is 3–5 days old, can give rise to all cell lineages that make up the human body.^[^ ^33^ ^]^ ESCs are highly valued for their potential for pluripotent differentiation and self‐renewal.^[^ ^11^ ^]^ During embryonic development, ESCs can form precursor cells that differentiate into nearly all cell types constituting the body when provided with appropriate signaling stimuli. However, inducing the lineage‐specific differentiation of ESCs under laboratory conditions remains challenging. Endodermal cells, such as stem cells, pancreatic islet *β* cells, and alveolar epithelial cells; mesodermal cells, such as osteocytes, chondrocytes, cardiomyocytes, and peripheral blood cells; ectodermal cells, such as keratinocytes; and various nerve cells have been successfully induced from ESCs.^[^ ^34^ ^]^ The differentiation potential of ESCs lays the foundation for their application in cell therapies for musculoskeletal diseases. Additionally, ESCs can self‐renew, meaning that cells can remain in an undifferentiated state during division and continue to divide into new stem cells. This characteristic has important implications for both the maintenance of ESC stemness and the perpetuation of the stem cell pool.^[^ ^35^ ^]^

Despite these advantages, the use of ESCs in cell‐based therapies remains challenging. Since harvesting ESCs requires destroying the embryo, there are medical and ethical restrictions on ESC‐related research in most countries. Much of the current debate surrounding human ESCs involves the ethical and legal dilemmas surrounding embryo destruction. Resolving this dilemma involves answering a series of philosophical questions, such as “when does life begin?” There is currently no clear consensus in academic circles. In addition, ESCs highly express cancer‐related genes, such as c‐Myc, which are essential for maintaining the stemness of ESCs, but also increase the possibility of tumor formation, thereby limiting the clinical application of ESC transplantation therapy.^[^ ^36^ ^]^

#### 3.2.3 iPSCs

iPSCs are stem cells created in a laboratory through the introduction of embryonic genes into somatic cells (e.g., skin cells), which then acquire pluripotent differentiation and self‐renewal potential similar to that of ESCs, while avoiding many of the limitations of ESCs.^[^ ^37^ ^]^ Shinya Yamanaka invented iPSC technology in 2006. They successfully converted murine fibroblasts into ESC‐like cell populations by introducing four specific genes (Oct3/4, Sox2, Klf4, and c‐Myc).^[^ ^38^ ^]^ Continuous efforts from researchers have made it possible to induce iPSCs from various human somatic cells, including keratinocytes, peripheral blood cells, and renal epithelial cells.^[^ ^39^, ^40^, ^41^, ^42^, ^43^, ^44^ ^]^

In 2013, Deng et al. successfully used small molecules to induce PSCs from mouse somatic cells.^[^ ^45^ ^]^ Later, in 2022, they revealed for the first time how human adult cells are converted into PSCs through four induction steps using a combination of more than 10 small‐molecule compounds.^[^ ^46^ ^]^ Compared to conventional approaches, small molecules offer straightforward operation, robust spatiotemporal modulation, reversible actions, and highly predictable cellular reprogramming. Small‐molecule inducer cell reprogramming also eliminates safety concerns associated with conventional transgenic procedures, such as tumorigenesis. Recently, Ding et al. effectively induced and maintained totipotent stem cells from mouse PSCs using a cocktail drug combination of three small compounds, TTNPB, 1‐azakenpaullone, and WS6, rather than transcription factors.^[^ ^47^ ^]^ With this combination, it was possible to transform mouse PSCs into cells that are most similar to the mouse 2C embryonic stage or stem cells with totipotent properties and to maintain them in culture. The small‐molecule chemical induction approach unquestionably expands the potential applications of iPSCs and offers a broader perspective for the stem cell and regenerative medicine industries.

#### 3.2.4 Muscle Stem Cells

Muscle stem cells, also known as myosatellite cells or satellite cells (SCs), are skeletal muscle‐specific progenitor cells located between the sarcolemma and basement membrane of muscle fibers. Satellite cells are considered powerful candidates for cell‐based therapies to treat muscle‐associated diseases because of their accessibility, self‐renewal capacity, and myogenic differentiation potential.^[^ ^48^ ^]^ Under normal physiological conditions, SCs remain quiescent. When exposed to activation signals resulting from exercise or injury, quiescent SCs are converted into activated SCs. The latter enter the cell cycle and begin to proliferate and differentiate, a process that plays an important role in initiating muscle regeneration.^[^ ^49^ ^]^ Some SCs differentiate into myoblasts to repair damaged muscles, whereas others proliferate into new SCs to replenish the stem cell pool.

Xu et al. isolated human SCs from muscle biopsies using fluorescence‐activated cell sorting and transplanted them into NOD *scid* gamma mice. They found that the transplanted cells stably engrafted, colonized the stem cell niche, and fused with host cells to form human‐derived myofibers.^[^ ^50^ ^]^ Muscles are the most abundant tissue in the human body, and the SCs within them are abundant and can be easily isolated from muscle biopsies. These properties increase the prospects for the clinical application of SCs.

#### 3.2.5 Skeletal Stem Cells

Human skeletal stem cells (SSCs) are a type of SSC with self‐renewal and pluripotent differentiation potential. Marecic et al. demonstrated the presence of SSCs in mice in 2015.^[^ ^51^ ^]^ In 2018, Chan et al. isolated human SSCs. SSCs typically differentiate into bone, cartilage, and stromal progenitor cells, which then differentiate into the bone, cartilage, and matrix. Following acute osteochondral injury, SSCs expand locally. The authors found that the expansion of human SSCs in cartilage callus specimens was significantly higher than that in uninjured bone tissue.^[^ ^52^ ^]^ Further research has revealed that the aging of SSCs alters the signaling of the stem cell niche and leads to the expression of high levels of pro‐inflammatory and pro‐resorptive cytokines, resulting in a weakened ability to generate bone and cartilage and poor bone regeneration.^[^ ^53^ ^]^ These findings demonstrate the potential of using SSCs in cell therapy.

### 3.3 Cell Derivatives and Organelles

#### 3.3.1 Exosomes

Exosomes are extracellular vesicles with a lipid bilayer membrane structure and are secreted by cells. They have diameters ranging from 30 to 150 nm.^[^ ^54^ ^]^ Exosomes are widely considered to be mediators of intercellular communication. They have been found to exert therapeutic effects in many animal models of tissue injury, including acute myocardial infarction, reperfusion injury, ischemic acute renal failure, wound healing, skeletal tissue repair, and acute kidney injury.^[^ ^55^, ^56^, ^57^, ^58^, ^59^ ^]^

Exosomes have garnered considerable attention for their potential use in cell‐based therapies because they offer several advantages. The immunogenicity of exosomes is extremely low due to exosome biogenesis, as described previously. When exogenous exosomes enter the body, they cause almost no immune response.^[^ ^60^ ^]^ As in the case of blood transfusions in hospitals, a large number of exosomes of exogenous origin can enter the body of a patient along with the blood but do not cause significant side effects. Furthermore, unlike stem cells, exosomes are not replicable and show no mutagenicity, which significantly reduces the possibility of tumorigenicity during treatment.^[^ ^61^ ^]^ This engineered approach allows a larger yield of exosomes, thereby laying the foundation for clinical development. Compared to stem cells, the preservation of exosomes is relatively simple and facilitates practical dissemination.

#### 3.3.2 Mitochondria

Spontaneous mitochondrial transfer occurs naturally and is thought to support tissue homeostasis. Gao et al. confirmed that aging reduces the distribution of mitochondria in primary osteocyte dendrites. They also discovered that intercellular mitochondrial transfer takes place inside the osteocyte dendritic network and that in stressed osteocytes without functional mitochondria, the transferred mitochondria restore cellular metabolism.^[^ ^62^ ^]^ Thus, promoting mitochondrial transfer between cellular networks may be a viable treatment for age‐related degenerative diseases, such as AD and osteoporosis. Research on intercellular mitochondrial transfer in the musculoskeletal system has primarily focused on chondrocytes, osteocytes, and skeletal muscles.^[^ ^62^, ^63^, ^64^ ^]^ Furthermore, research on mitochondrial therapy may promote the development of stem cell therapies. As undifferentiated stem cells are often used for mitochondrial donation and are believed to enhance the therapeutic effect of stem cells, further investigation is needed to understand the mechanisms that promote transfer efficacy, such as increasing the formation of transfer routes or strengthening factors that facilitate mitochondrial motility.^[^ ^65^, ^66^ ^]^

## 4 Attempts to Use Cell‐Based Therapies in the Treatment of DMDs

### 4.1 Cell‐Based Therapy for Arthritis

OA manifests as damage to and insufficient regeneration of the cartilage owing to factors, such as stress or aging. Low‐grade chronic inflammation around the joints, such as synovial inflammation, may promote symptoms and accelerate the progression of OA. Cell therapy for OA aims to regenerate the cartilage, inhibit inflammation, and restore the area around the joint to a normal physiological state.

#### 4.1.1 Chondrocyte Transplantation

The clinical efficacy of autologous chondrocyte implantation (ACI) has been previously demonstrated. ACI was performed in patients with knee cartilage lesions, and good therapeutic effects were observed for up to 10 years post‐surgery.^[^ ^67^ ^]^ The procedure consists of two steps. First, a needle biopsy is performed to obtain cartilage tissue from the non‐weight‐bearing area of the joint. These tissues are digested into isolated chondrocytes that undergo proliferation and expansion. The expanded cells are implanted into the areas of defective cartilage through a second operation and covered periosteally to seal the defect.^[^ ^68^ ^]^ The most common complication after ACI is excessive hypertrophy of the periosteal flap used to seal the implanted chondrocytes.^[^ ^69^ ^]^ Additionally, in the process of cartilage transplantation, other problems can arise, such as uneven distribution of chondrocytes and possible leakage of cell suspensions from cartilage lesions. Chondrocytes cultured in vitro are more easily dedifferentiated and display fibroblast markers, which reduce the purity of ACI grafts and may cause engraftment failure.

Different strategies, including matrix‐induced ACI (MACI), have been used to improve ACI surgery.^[^ ^70^ ^]^ MACIs are similar to ACIs. The difference between them is that MACIs use artificial scaffolds, such as porcine membranes consisting of a mixture of collagens, to assist in cell culture and expansion after chondrocytes are isolated. These membranes are specifically designed to have low friction on one side and favor chondrocyte ingrowth. During surgery, the smooth surface faces the articular cavity, and the cell‐laden surface faces the subchondral bone. Fiber glue can be used for implantation to avoid the use of watertight sutures. MACIs have an advantage over ACIs because problems, such as uneven cell distribution and cell leakage, can be avoided.^[^ ^71^ ^]^ The operation time is also greatly shortened because there are no periosteal removal or suturing steps. A randomized controlled study showed that the MACI‐treated group had a lower probability of graft hypertrophy than the ACI‐treated group, although the clinical and histological outcomes were comparable.^[^ ^72^ ^]^ Further studies are required to clarify the outcomes of both treatments.

However, both ACIs and MACIs have drawbacks. First, patients are required to undergo two surgical interventions: chondrocyte harvesting and cartilage implantation. This inevitably causes two surgical traumas, which may be unacceptable for patients whose physical conditions do not allow or tolerate trauma. Second, the cycle of treatment and rehabilitation is long and usually takes 6–12 months to ensure the maturation of new tissue and to obtain a good therapeutic effect.^[^ ^73^ ^]^ Ensuring patient compliance is difficult during this process. In addition, if a patient suffers from other injuries, the effect of treatment can be greatly attenuated. Therefore, more efficient treatment methods and better therapeutic schemes are required to treat cartilage lesions in diseases such as OA.

#### 4.1.2 Attempts to Use MSCs for the Treatment of Arthritis

MSCs play a unique role in OA therapy. The major therapeutic mechanisms include the direct differentiation of MSCs into chondrocytes and their paracrine effects, which skew the local pro‐inflammatory microenvironment for tissue healing. Members of the transforming growth factor superfamily (e.g., TGF‐*β*1, TGF‐*β*2, and TGF‐*β*3) and several growth factors (e.g., IGF‐1, BMP‐6, FGF‐2, epidermal growth factor, and PDGF) are commonly used to induce the differentiation of MSCs into chondrocytes.^[^ ^74^ ^]^ Animal studies have suggested that MSCs can reduce cartilage destruction and subchondral bone remodeling and promote cartilage regeneration.^[^ ^75^, ^76^, ^77^ ^]^ Murphy et al. delivered adult MSCs to caprine knee joints following the induction of OA and observed meniscal tissue regeneration, which is believed to delay the progressive destruction normally observed in this OA model.^[^ ^78^ ^]^ Diekman et al. isolated MSCs from bone marrow, injected them into the articular space and verified that MSC therapy inhibited OA progression in a mouse model.^[^ ^79^ ^]^ Adipose‐derived stem cells have also been used to treat experimentally induced OA in rabbits. The experimental group showed better cartilage quality after treatment than the control group.^[^ ^80^ ^]^ An inflammatory environment, especially with the presence of inflammatory cytokines, such as TNF‐*α*, IL‐1*β*, and IL‐6, can inhibit the formation of new cartilage. MSCs can reduce inflammation around joints by attenuating inflammatory cytokines, such as IL‐1*β*. In addition, MSCs inhibit the activation of M1 macrophages, promote their transformation to the M2 phenotype, suppress the proliferation and cytotoxicity of NK cells, restrain the production of B‐cell antibodies, activate CD4^+^ T cells, and promote the production of immunosuppressive CD4^+^ T regulatory cells (Tregs).^[^ ^81^ ^]^ These immunosuppressive features of MSCs can reduce the occurrence of graft‐versus‐host disease following allogeneic transplantation.

#### 4.1.3 Attempts to Use iPSCs for the Treatment of Arthritis

The chondrogenic potential of iPSCs provides a new direction for the use of cell‐based therapies in the treatment of OA. Their use circumvents the ethical issues associated with ESCs. However, there is currently no perfect protocol for iPSC induction. Csobonyeiova et al. summarized four main approaches for chondrogenic differentiation: 1) induction of iPSCs to first differentiate into MSCs and then into chondrocytes, 2) coculturing iPSC‐derived MSCs with primary chondrocytes, 3) differentiation of chondrocytes through embryoid bodies (EBs), and 4) improving the media for iPSCs by mimicking the cartilage growth environment. Growth factors, such as TGF‐*β*, BMP, WNT3A, and FGF‐2, and paracrine factors, such as Ihh and Runx, play important roles in shifting cell fate toward the chondrogenic lineage.^[^ ^82^ ^]^ An indirect differentiation approach based on the EBs is classic and reliable. However, newer protocols for inducing direct chondrogenic differentiation of iPSCs have been developed in recent years. These protocols involve the addition of exogenous mesodermal and chondrogenic growth factors and the utilization of conditioned media, which are more efficient and cost‐effective methods.^[^ ^83^ ^]^ However, additional research is needed to better understand the nature of the chondrogenic differentiation of iPSCs.

Although there are feasible schemes for the differentiation of iPSCs into chondrocytes, it is difficult to generate cartilage tissues. Cartilage is not only composed of chondrocytes but also contains a large amount of ECM, proteoglycans, and elastins. Therefore, the use of iPSCs to achieve cartilage regeneration usually requires a combination of tissue engineering methods. Yamashita et al. used a scaffold‐free suspension culture supplemented with BMP2, TGF‐*β*1, and GDF5 to generate cartilage particles in vitro. The cultures were implanted into immunodeficient mice with damaged joints. New cartilage formation was observed 12 weeks after transplantation. In addition, no tumors or ectopic tissues were generated after transplantation.^[^ ^84^ ^]^ Many researchers have sought to cure OA by transplanting iPSC‐derived chondrocytes or cartilage into articular cartilage defects, and some have shown promising outcomes.^[^ ^85^ ^]^ However, the safety of this method requires careful evaluation. Long‐term observations of the healing of large defects in large animals are still desirable to evaluate the efficacy of transplantation therapy in preclinical studies. In addition, most current research has focused on improving cartilage conditions, with few studies focusing on relieving pain symptoms, which is critical when addressing clinical problems.

#### 4.1.4 Attempts to Use Exosomes and Mitochondria for the Treatment of Arthritis

Several preclinical studies have confirmed the effectiveness of exosomes in the in vivo treatment of OA. MSC exosomes have been extensively studied and found to repair cartilage damage and mediate cartilage regeneration. For example, human‐derived MSC exosomes effectively alleviated cartilage damage and matrix degeneration in a mouse model of OA by increasing collagen type II levels and decreasing the expression of ADAMTS5 in the presence of IL‐1*β*.^[^ ^86^ ^]^ Exosomes produced by chondrocytes can act on chondrocyte progenitor cells and bone marrow‐derived MSCs (BM‐MSCs) to promote chondrogenic differentiation and chondrogenesis via activation of the Wnt/*β*‐catenin signaling pathway by exosomal miR‐8485.^[^ ^87^ ^]^ Tao et al. found that miR‐140‐5p‐overexpressing exosomes extracted from the synovial tissue can act on articular chondrocytes, increase chondrocyte proliferation and migration, promote cartilage regeneration, maintain the ECM of the cartilage.^[^ ^88^ ^]^ Exosomes produced by MSCs in the infrapatellar fat pad exert a protective effect on cartilage by inhibiting the mTOR pathway.^[^ ^89^ ^]^ Many ncRNAs carried by exosomes are thought to play important roles in this process. For example, miR‐140‐5p is involved in the Wnt signaling pathway and regulates the expression of type I and II collagen.^[^ ^88^ ^]^ miR‐92a‐3p promotes chondrogenesis and suppresses cartilage degradation.^[^ ^90^ ^]^

Exosomes also mediate extensive intercellular communication among the component cells within the joint, including chondrocytes, synovial cells, osteocytes, fibroblasts, and immune cells. Understanding the physiological and pathological roles of exosomes in cartilage destruction and regeneration may provide guidelines for the application of exosomes from various sources for the treatment of arthritis. For instance, exosomes from primary chondrocytes cultivated in a normal environment (D0 exosomes) may correct mitochondrial dysfunction and increase M2 macrophage penetration, while decreasing M1 macrophage penetration. Intra‐articular administration of D0 exosomes effectively slowed the development of OA.^[^ ^91^ ^]^ Exosomes produced by the synovium, ligaments, and subchondral bone are involved in regulating joint inflammation, injury repair, and other processes.^[^ ^92^ ^]^ Nonetheless, further investigation into the mechanisms of exosome generation and release in the joint is required, and there is still a lack of direct evidence that endogenous exosomes can be transferred from one cell to another in the joint in vivo. Studying these issues in depth may help researchers better dissect the exosome‐related mechanisms involved in maintaining cartilage integrity.

Previous studies have reported that mitochondrial dysfunction participates in the pathological process of OA. Wang et al. harvested and cocultured BM‐MSCs and OA chondrocytes. They verified that mitochondria could be transferred from BM‐MSCs to chondrocytes.^[^ ^63^ ^]^ By enhancing mitochondrial activity and cell proliferation and suppressing apoptosis in chondrocytes, they further demonstrated that mitochondrial transfer could protect OA chondrocytes against mitochondrial dysfunction and degeneration. This discovery may present a novel therapeutic approach for OA.

### 4.2 Cell‐Based Therapy for Sarcopenia

Sarcopenia is an age‐related condition. Age‐associated factors, including inflammation, play an important role in the pathogenesis of sarcopenia.^[^ ^93^ ^]^ Since mature muscle cells cannot undergo mitosis, stem cells are the only promising candidates for muscle regeneration. MSCs, iPSCs, SCs, and exosomes have been used as therapeutic agents for sarcopenia because of their regenerative abilities.

#### 4.2.1 Attempts to Use MSCs for the Treatment of Sarcopenia

Chronic inflammation is a major contributor to the development of sarcopenia. Aged muscle cells release chronic inflammatory factors, leading to an inflammatory environment around muscle cells.^[^ ^94^ ^]^ The accumulation of reactive oxygen radicals due to old age also plays an important role in the pathogenesis of sarcopenia. Palomero et al. used a nonspecific fluorescent probe to directly assess reactive oxygen species (ROS) activity in skeletal muscle.^[^ ^95^ ^]^ Owing to their anti‐inflammatory properties, MSCs may be outstanding candidates for cell‐based therapies to attenuate sarcopenia. MSCs can convert a pro‐inflammatory environment into an anti‐inflammatory environment, contributing to the proliferation and function of SCs.^[^ ^96^, ^97^ ^]^ Additionally, growth factors secreted by MSCs, such as FGF, VEGF, and monocyte chemotactic protein 1, have neuroprotective functions.^[^ ^98^ ^]^ Therefore, MSCs can facilitate muscle regeneration by restoring endogenous cell activity and reinnervating the muscle tissue.

#### 4.2.2 Attempts to Use Satellite Cells for the Treatment of Sarcopenia

Satellite cells (SCs) are important for muscle regeneration. The number of SCs decreases with sarcopenia.^[^ ^99^ ^]^ Exercise, which is considered the most effective means of treating sarcopenia, restores the number of SCs. Aged SCs are more prone to apoptosis and senescence than young cells. In addition, aging of the immune system leads to a decrease in muscle stem cell populations in elderly people. Several signaling pathways, such as Notch, JAK/STAT, p38 MAPK, and Wnt, have been found to link SCs to sarcopenia.^[^ ^100^ ^]^ Collins et al. found that transplanting even a small number of SCs could generate large numbers of new myoblasts. Transplanted SCs also have the ability to self‐renew and maintain a stable endogenous stem cell pool.^[^ ^101^ ^]^

In addition to SCs, stem cells can be integrated into tissue engineering materials, such as scaffolds, to promote muscle regeneration. Researchers have successfully applied tissue engineering methods to generate muscle fibers in vitro and achieve local muscle regeneration through transplantation.^[^ ^102^ ^]^ Natural and artificial materials, such as fibrin, alginate, and polycaprolactone (PCL)‐based polymers, have been used to generate skeletal muscle tissue in vitro. However, despite significant advancements in skeletal muscle tissue engineering, fully functional skeletal muscle tissue constructs are yet to be created in vitro. These tissue engineering approaches have a limited ability to induce precise 3D spatial cell arrangements. 3D printing technology, which has been developed in recent years, can achieve a high‐precision distribution of cells and matrices to rapidly fabricate complex structures, such as skeletal muscle tissue.^[^ ^103^ ^]^ For example, Gao and Cui revealed a bioprinting approach that can be utilized to accurately deposit mouse myoblasts (C2C12) into a matrix on cantilevers with a precision of 85 µm, >90% cell survival, and good reproducibility.^[^ ^104^ ^]^ This novel technique has expanded the applicability of methods used in muscle regeneration research.

#### 4.2.3 Attempts to Use Exosomes and Mitochondria for the Treatment of Sarcopenia

The therapeutic role of exosomes in sarcopenia has been gradually recognized. Myogenesis is precisely regulated by the body and relies on coordinated intercellular interactions, in which exosomes play an important role. Exosomes released by skeletal muscle have been revealed to not only enhance myoblast proliferation and myogenesis but also convey signaling molecules across muscle cells, which indicates the interaction of exosomes between myocytes and myoblasts. According to Aswad et al., feeding mice a high‐fat diet causes their skeletal muscles to release exosomes, which then stimulate myoblast proliferation and alter the expression of genes that control the muscle cell cycle and differentiation in vitro.^[^ ^105^ ^]^ Muscle‐derived exosomes not only act on muscle cells but also induce the proliferation, migration, and formation of human umbilical vein endothelial cells (ECs). Additionally, exosomes promote NSC‐34 motor neuron cell survival and neurite outgrowth. This indicates that muscle‐derived exosomes may also play a role in neurogenesis and angiogenesis.^[^ ^106^, ^107^ ^]^ Exosomes derived from other cells can also act on muscle cells. For example, exosomes derived from MSCs promote the proliferation and differentiation of C2C12 cells, while adipocyte‐derived exosomal miR‐27a induces insulin resistance in skeletal muscle cells.^[^ ^108^ ^]^

Research on the use of exosomes in the treatment of muscle diseases is still in the early stages of development. Skeletal muscle cells are rich in various miRNAs, such as miR‐31 and miR‐23a, which are thought to regulate SC activation, promote muscle regeneration, and reduce muscle atrophy. These miRNAs can be secreted into target cells via exosomes, indicating their potential use in the treatment of muscle diseases. At the therapeutic level, exosomes produced by human skeletal myoblasts and MSCs have been found to accelerate muscle regeneration.^[^ ^109^, ^110^ ^]^ For example, MSC‐derived exosomes promote the proliferation and differentiation of C2C12 cells in skeletal muscles, which are partially mediated by miR‐494.^[^ ^110^ ^]^ Another study found that exosome therapy promoted the development of human muscle cells by transferring exosomal miR‐29c. Furthermore, MSCs generated from the placenta release exosomes with high miR‐29 expression.^[^ ^111^ ^]^ These results highlight the enormous potential of exosomes as therapies for muscle disorders.

Mitochondrial dysfunction is a pathogenic factor in sarcopenia. According to a recent study, increased generation of mitochondrial ROS is a significant factor in mitochondrial malfunction and damage linked to protracted inactivity in the skeletal muscle. Recent studies have demonstrated that healthy or modified mitochondria can be transported to diseased cells, thereby restoring cellular functions. According to Kim et al., mitochondrial transfer restores ATP synthesis in myoblast cells, mitochondrial membrane potential, ROS, and oxygen consumption rate to normal.^[^ ^64^ ^]^ Additionally, in atrophic muscle cells, the introduction of undamaged mitochondria prevented the AMPK/FoxO3/atrogene pathway that causes muscular atrophy. Collectively, these straightforward mitochondrial transfer procedures can be used to treat sarcopenia.

### 4.3 Cell‐Based Therapy for DDDs

IVDs are composed of a central hydrophilic proteoglycan‐rich nucleus pulposus (NP), which is surrounded by an annulus fibrosus (AF) and cartilaginous and bony endplates that separate the discs from the vertebrae. Normally, the anabolic and catabolic activities of the IVD cells are balanced. However, this balance can be disrupted under pathological conditions, such as excessive stress, which leads to a degenerative cascade characterized by alterations in the ECM, metabolic dysregulation of NP cells, inflammation, and oxidative stress.^[^ ^112^ ^]^ Cell therapy for DDD aims to restore the normal physiological environment and function of IVDs.

#### 4.3.1 Direct Transplantation of IVD Cells

Autologous NP cell transplantation is another strategy used to treat DDDs. In degenerated IVDs, collagen synthesis in NP cells changes, with a decrease in type II collagen synthesis and an increase in type I collagen synthesis, leading to a reduction in the elasticity and mechanical properties of the IVD tissue and the ability of the IVDs to bear loads.^[^ ^113^ ^]^ Theoretically, NP cell transplantation can replace degenerated NP cells. The safety of this technique was confirmed in a 3‐year clinical trial.^[^ ^114^ ^]^ The researchers further demonstrated the minimal efficacy required to slow the further degeneration of human IVDs. Additionally, the low cell density and proliferative activity of autologous NP cells during the early stages of primary culture were resolved by coculturing the cells in direct contact with MSCs in vitro.^[^ ^114^ ^]^ However, the results of an efficacy evaluation in a phase I clinical trial were unsatisfactory.^[^ ^114^ ^]^

AF cell transplantation is another strategy used to treat DDDs. AF cells are elongated mesenchymal fibroblasts with cytoplasmic protrusions. They can synthesize proteoglycans and collagen to form annular lamellae that protect the NP. Researchers have attempted to use AF cells for transplantation to provide a more biocompatible environment and improve NP cell function. Sato et al. cultured AF cells from rabbit IVDs in an atelocollagen scaffold. After in vitro culture for 7 days, AF cells were transplanted into the lacunae of the IVDs. Researchers have found that transplanted AF cells remain viable and show proliferative activity.^[^ ^115^ ^]^ When AF cells were transplanted into a recipient rabbit in which the NP had been vaporized using an indocyanine green dye‐enhanced laser, the narrowing of the IVD space in the cell transplantation group was significantly reduced.^[^ ^116^ ^]^

Several studies have used biomaterials to implant NP and AF cells simultaneously for DDD treatment. Xu et al. designed and fabricated a biomimetic biphasic scaffold for IVD engineering in which pig bone matrix gelatin was used for the outer AF phase and pig acellular cartilage ECM was used for the inner NP phase. The matrix was implanted subcutaneously in nude mice after successful observation of fluorescently labeled NP and AF cells anchored on the scaffolds. Six weeks after implantation, IVD‐like tissues were formed in nude mice, as confirmed by histological analysis.^[^ ^117^ ^]^ This study demonstrated that the use of biomaterials, such as biphasic scaffolds, may be a future direction for cell‐based DDD therapy.

There are several obstacles to the direct transplantation of IVD cells. The IVD niche has many features, such as avascularity, low cellularity, high mechanical stress, high osmotic pressure, and low pH. Despite increasing evidence from preclinical studies in animal models or in vitro studies of human cells, further studies are needed to determine whether the transplanted cells can adapt and function for a reasonable time in the setting of progressive degeneration within the exceptional “niche” of human IVDs. Furthermore, obtaining viable donor IVD cells from autologous sources is problematic because obtaining cells from a healthy IVD may result in iatrogenic IVD injury, and cells separated from degenerated IVD tissues may be insufficiently viable or functional.^[^ ^118^ ^]^ Overcoming these limitations may facilitate the clinical application of IVD cell transplantation.

#### 4.3.2 Attempts to Use Stem Cells for the Treatment of DDDs

Direct differentiation of MSCs into NP cells has not yet been achieved, as there is currently no well‐established protocol. Richardson et al. induced MSCs in chitosan–glycerophosphate hydrogels to differentiate into cells with a phenotype similar to that of articular chondrocytes and NP cells.^[^ ^119^ ^]^ NP cells are similar to chondrocytes and express chondrocyte markers, such as SOX‐9, type II collagen, and aggrecan.^[^ ^120^ ^]^ However, injecting such cells into the NP does not result in the appropriate hydrogel matrix characteristics of the NP, but instead results in the generation of hyaline cartilage. Therefore, transplantation of cells with a cartilage‐like phenotype is not the best option for repairing damaged discs.^[^ ^121^ ^]^

MSCs can communicate with NP cells and affect their function through bioactive molecules, such as anabolic growth factors. Strassburg et al. demonstrated that MSCs could communicate with NP cells via cell fusion, gap junction communication, and exchange of membrane components via direct transfer or microvesicle formation.^[^ ^122^ ^]^ Miyamoto et al. transplanted allogeneic synovial MSCs into the NP space in a rabbit model of disc degeneration. They found that NP cells increased collagen type II synthesis and suppressed the expression of inflammatory cytokines and degrading enzymes, thereby protecting the remaining disc structures.^[^ ^123^ ^]^

ESCs and iPSCs can differentiate into chondrocytes and NP cells. Some researchers believe that the effects of treatment with non‐mesenchymal lineage cells on IVDs are limited, and few studies have assessed the use of iPSCs for the treatment of DDDs.^[^ ^124^ ^]^ One study used NP cell‐derived iPSCs for NP induction. The results showed that NP cell‐specific matrix proteins and related genes were expressed and that NP‐derived iPSCs differentiated much better in the hydrogel than in the culture plate.^[^ ^125^ ^]^

Most studies on stem cell differentiation into IVD cells have focused on the NP cell phenotype. In contrast, other cells, such as AF or notochordal cells, have received less attention. Nevertheless, the repair of the AF may be of great use in reversing the effects of herniations and tears, which result in prolapsed or herniated “slipped” discs. Notochordal cells have been found to play a protective role against disc degeneration. Thus, further studies are required to develop methods for inducing stem cells toward IVD component cells other than NP and to evaluate the therapeutic effects of these cells in the treatment of DDDs.

#### 4.3.3 Therapeutic Function of Exosomes

Several studies have reported the regulatory role of exosomes in the pathogenesis of DDDs. NP cell‐derived exosomes can promote MSC migration, downregulate the Notch1 signaling pathway, and reduce inflammation through the NF‐*κ*B signaling pathway.^[^ ^126^, ^127^ ^]^ Notochord cell‐derived exosomes have been found to increase the DNA and glycosaminoglycan content in NP cells.^[^ ^128^ ^]^ Furthermore, multiple in vivo and in vitro studies have explored the possibility of using exosomes to treat DDDs. As found in in vitro experiments, human BM‐MSC‐derived exosomes can reduce the secretion of IL‐1*β*, activate the MAPK signaling pathway through miR142‐3P, reduce the apoptosis of IVD cells, and promote their proliferation.^[^ ^129^ ^]^ In vivo, studies have attempted to treat IVDD using exosomes. In a rabbit model of IVDD, exosomes diminished IVDD progression by inhibiting NLRP3 inflammasome formation and restoring damaged mitochondria.^[^ ^130^ ^]^ In a rat model of IVDD, BM‐MSC‐derived exosomes ameliorated NP cell apoptosis by activating the PI3K‐AKT pathway via miR‐21.^[^ ^131^ ^]^ Exosomes from iPSC‐derived MSCs and normal cartilage endplate stem cells have also been found to delay the progressive degeneration of IVDs.^[^ ^132^, ^133^ ^]^

### 4.4 Cell‐Based Therapy for Osteoporosis

Osteoporosis is characterized by an imbalance in the bone remodeling process in which osteoclast‐mediated bone resorption exceeds osteoblast‐mediated bone formation, eventually leading to bone loss and microarchitectural destruction. Various factors may contribute to imbalances in bone remodeling, such as estrogen deficiency, aging, inflammation, and vitamin D. Cell‐based therapy for osteoporosis aims to restore normal bone remodeling through mechanisms that promote bone formation, inhibit bone resorption, and eliminate pro‐imbalance factors.

#### 4.4.1 Attempts to Use MSCs for Anti‐Osteoporosis Therapy

The original rationale for using MSCs as anti‐osteoporosis agents was their osteogenic differentiation potential. Jaiswal et al. first established a reproducible system for in vitro osteogenic differentiation of human MSCs.^[^ ^134^ ^]^ Since then, preclinical studies have demonstrated the effectiveness of MSC therapy for inducing bone formation and treating osteoporosis. Hsiao et al. injected MSCs into mice with ovariectomy (OVX)‐induced osteoporosis. Two months after injection, the bone density and volume of the OVX mice were restored to normal levels.^[^ ^135^ ^]^ To increase delivery efficiency, Guan et al. developed a method to direct MSCs to the bone surface by attaching a synthetic high‐affinity and specific peptidomimetic ligand (LLP2A) against integrin *α*4*β*1 on the MSC surface to a bisphosphonate that had a high affinity for bone. A single dose of LLP2A‐Ale MSCs prevented trabecular bone loss induced by estrogen deficiency.^[^ ^136^ ^]^

Since the first observation of the anti‐osteoporotic effects of MSCs, several mechanisms of action have been identified. Wang et al. transplanted primary MSCs into OVX mice and demonstrated that they effectively prevented postmenopausal osteoporosis development by promoting intraosseous angiogenesis, which is involved in the reestablishment of microcirculation within the skeleton.^[^ ^137^ ^]^ Furthermore, researchers found that the coculture of MSCs with osteoclast precursors can significantly reduce the ability of the precursors to differentiate into mature osteoclasts. During this process, the expression of osteoprotegerin (OPG) by MSCs is significantly elevated.^[^ ^138^ ^]^ OPG is a decoy receptor for the receptor activator of nuclear factor kappa‐B ligand (RANKL), which stimulates osteoclast differentiation and function. Upon binding to RANKL, OPG prevents it from interacting with receptors on the surface of osteoclast precursors, such as RANK, thereby inhibiting osteoclast maturation. These findings indicate that MSCs not only differentiate into osteoblasts but also exert a negative effect on osteoclast differentiation.

#### 4.4.2 Attempts to Use iPSCs for Anti‐Osteoporosis

The osteogenic differentiation potential of iPSCs has been confirmed both in vivo and in vitro, although it is not as efficient as that of MSCs. Tashiro et al. introduced a transduction method that successfully induced iPSC differentiation into osteoblasts via exogenous overexpression of Runx‐2.^[^ ^139^ ^]^ Yu et al. used retinoic acid to induce the rapid osteogenic differentiation of iPSCs in vitro. They found that this process could be improved using 3D‐printed Ti6Al4V scaffolds.^[^ ^140^ ^]^ In vivo, experiments have confirmed the ability of iPSCs to form bones. Duan et al. combined enamel matrix derivatives (EMDs) with human iPSCs for periodontal tissue regeneration in nude mice. Histological analysis showed more new alveolar bone and cementum formation in the iPSC‐treated group than in the EMD controls.^[^ ^141^ ^]^ In addition, iPSCs can repair long bone segmental defects, and iPSC‐derived MSCs have been demonstrated to form new bones to repair cranial bone defects.^[^ ^142^ ^]^ However, evidence on the application of iPSCs in the treatment of osteoporosis is lacking. Moreover, the in situ differentiation of iPSCs into osteoblasts or even MSCs remains challenging and hinders the application of iPSCs in this scenario. A possible way to overcome this challenge may be to induce iPSCs to differentiate into osteoblasts in vitro and then deliver these osteoblasts to the osteoporosis lesion site. Further investigations are required to determine the efficacy of this strategy.

#### 4.4.3 Attempts to Use Exosomes for Anti‐Osteoporosis

Physiologically, exosomes play an important role in maintaining bone homeostasis by mediating communication among executive cells within bone tissue.^[^ ^143^ ^]^ For example, Wang et al. found that miR‐214 may play an inhibitory role in the regulation of bone formation.^[^ ^144^ ^]^ Later, in 2016, Li et al. found elevated osteoclast exosomal miR‐214 levels in the sera of elderly women with fractures and verified their association with reduced osteogenesis. Osteoclast‐derived exosomal miR‐214‐3p indicated that exosomes are key mediators of homeostatic regulation.^[^ ^145^ ^]^ MSC‐derived exosomes promote osteoblast proliferation, inhibit apoptosis, and promote osteoblast differentiation and matrix calcification. Studies have indicated that monocyte‐derived exosomes can induce osteogenic differentiation of MSCs.^[^ ^146^ ^]^ Osteoblasts also secrete exosomes that promote osteogenic differentiation of MSCs and pre‐osteoblasts in an autocrine manner.^[^ ^147^ ^]^ Osteoblasts inhibit osteoclast activity and induce osteoclast apoptosis by secreting exosomes containing miRNAs, such as miR‐503‐3p and miR‐214‐3p.^[^ ^148^, ^149^ ^]^ In addition to bone executive cells, ECs can secrete exosomes that influence bone remodeling. EC‐derived exosomes, which express high levels of miR‐155, can be transferred into bone marrow‐derived macrophages and subsequently inhibit their differentiation into osteoclasts.^[^ ^150^ ^]^

The anti‐inflammatory and immunomodulatory functions of exosomes have been previously reported. MSC‐derived exosomes increase the transition from M1 to M2 macrophages, which are thought to have immunosuppressive and tissue‐repairing properties.^[^ ^151^ ^]^ Exosomes are also involved in regulating T‐cell activity. Du et al. demonstrated that MSC‐derived exosomes enhanced the anti‐inflammatory capacity of Tregs. They found that anti‐inflammatory cytokines, such as IL‐10 and TGF‐*β*1, may be important in this process.^[^ ^152^ ^]^ The accumulation of Th17 cells has been previously reported to be associated with osteoporosis because they secrete TNF‐*α* and IL‐7, thereby promoting osteoclast formation and activation.^[^ ^153^ ^]^ Chen et al. found that MSC‐derived exosomes increased the plasticity of Th1/Th2 cells and decreased the Th17 differentiation of peripheral blood mononuclear cells.^[^ ^154^ ^]^ These findings suggest that stem cell‐derived exosomes alter the immune compartment systemically and within the bone, thus, affecting the balance of bone remodeling.

Understanding the physiological functions of exosomes may further facilitate the application of exosomes derived from various cells to achieve the anticipated goals in osteoporosis treatment. Qi et al. found that MSC‐derived exosomes stimulated bone regeneration in OVX rat models with calvarial defects.^[^ ^155^ ^]^ MSC‐derived exosomes stimulated osteoblast development, activity, and proliferation in vivo mostly through miR‐196a.^[^ ^156^ ^]^ EC‐derived exosomes have been found to be superior in targeting bones and substantially suppress osteoclast development and activity in vitro and in animal studies. Therefore, EC‐derived exosomal miR155, miR‐503, miR133a, and miR‐422a may have the potential for the development of drugs against osteoporosis.^[^ ^157^ ^]^

As mentioned above, exosomes are relatively easy to manipulate through surface or content modifications, and a well‐designed engineering strategy to augment their function and increase their delivery efficiency to lesions may be adopted to achieve better results in the treatment of targeted diseases. Hu et al. developed a bone‐targeting delivery approach by engineering exosomes for the treatment of osteoporosis. Lentiviral transfection was used to introduce CXCR4 into NIH‐3T3 cells and obtain exosomes overexpressing CXCR4. CXCR4^+^ exosomes were then fused to liposomes containing antagomir‐188. Because of the surface CXCR4 molecules, these exosome–liposome hybrid nanoparticles specifically accumulated in the bone marrow and released antagomir‐188, which stimulated osteogenesis and inhibited adipogenesis in BM‐MSCs, thereby reversing age‐related trabecular bone loss and decreasing cortical bone porosity in mice.^[^ ^158^ ^]^ Cui et al. constructed an engineered exosome delivery system, BT‐Exo‐siShn3, based on the exosomes of MSCs derived from iPSCs. The bone‐targeting peptide modified with a diacyl lipid tail was anchored to the exosome membrane via hydrophobic interactions. The exosomes were then loaded with siRNA of Shn3 via electroporation. After injecting these engineered exosomes into osteoporotic mice, researchers found the promotion of osteoblast function and suppression of osteoclast activity, indicating anti‐osteoporosis effects.^[^ ^159^ ^]^

Cell therapy has yielded promising results for DMDs. The main characteristic of these cell therapy protocols is their ability to act therapeutically by altering cell differentiation, trophic function, and immunological modulation. Cell therapy is intended to completely eradicate a disease by reversing its pathogenic mechanisms. This differs from the current clinical treatment strategy, which focuses mainly on reducing pain and delaying illness progression. Although some studies have been clinically deployed and have demonstrated positive outcomes, many research programs are still in the exploratory phase.

## 5 Clinical Trials for Cell‐Based Therapies

Preliminary clinical trials have focused on the safety of stem cells for the treatment of degenerative diseases of the musculoskeletal system (**Table** **1**). Several ongoing clinical trials are aimed at clarifying the safety and efficacy of cell‐based therapies (**Table** **2**).

**Table 1:** Completed and published clinical trials investigating cell‐based therapies for OA, osteoporosis, and DDDs

Indication (stage)	Cell type (source)	Dosage and usage	No. of Subjects	Duration [Months]	Delivery Methods	Clinical Measure	Clinical Effect	Ref (Trial ID)
OA, Knee (Kellgren‐Lawrence grade 2 to 4)	AD‐MSCs (autologous)	10^8^ cells in 3 ml of saline (MSC group)	24	6	IA injection	Improvement of the WOMAC score; no serious adverse events; no significant change in the cartilage defect	Injection provides functional improvement and pain relief	Lee et al.^[^ ^160^ ^]^ (NCT02658344)
OA, Knee (late‐stage Kellgren–Lawrence)	BM‐MSCs	1, 10, 50 million cells in each group	12	12	IA injection	No serious adverse events; better than the baseline level	50 million doses achieved clinically relevant improvements	Chahal et al.^[^ ^161^ ^]^ (NCT 0 235 1011)
OA, Knee (grade 1–3 Kellgren–Lawrence)	UC‐MSCs	single‐dose(20 × 10^6^) versus repeated doses (20 × 10^6^ × 2)	29	12	IA injection	No severe adverse events; repeated UC‐MSC treatment is safe	Repeated UC‐MSC treatment is safe and superior	Matas et al.^[^ ^162^ ^]^ (NCT 0 258 0695)
OA, Knee (Kellgren–Lawrence grade 2 to 4)	BM‐MSCs (autologous)	10 × 10^6^ or 100 × 10^6^ (BM‐MSCs)	27	12	IA injection	No severe adverse events; BM‐MSC treatment is safe	Injection of BM‐MSCs is safe and feasible	Lamo et al.^[^ ^163^ ^]^ (NCT 0 212 3368)
OA, Knee (grade 1–3 Kellgren–Lawrence)	Placenta‐derived MSCs	0.5–0.6 × 10^8^ cells (MSC group)	20	6	IA injection	Improvements in quality of life, activity of daily living	Injection of BM‐MSCs is safe and feasible	Khalifeh et al.^[^ ^164^ ^]^ (N/T)
OA, Knee (Kellgren–Lawrence grade 2 to 4)	BM‐MSCs (autologous)	40 × 10^6^ cells (MSCs group)	43	6	IA implantation	Determination of the safety and efficacy of autologous MSCs in OA	Injection of BM‐MSCs is safe and feasible	Emadedin et al.^[^ ^165^ ^]^ (N/T)
OA, Knee (Kellgren–Lawrence grade 2 to 3)	AD‐MSCs (autologous)	100 × 10^6^ ADMSCs	30	6	IA injection	No serious adverse events; a safe and effective therapy for knee OA	Injection of BM‐MSCs is safe and feasible	Freitag et al.^[^ ^166^ ^]^ (ACTRN12614000814673)
OA, Knee (Kellgren–Lawrence grade 2 to 3 and planned to undergo TKA)	BM‐MSCs (autologous)	bone marrow graft of 20 cm^3^ volume (10 cc in tibia and 10 cc in femur) contained average of 7800 MSCs mL^−1^ (range 3120 to 11560)	140	180	IA injection	Sufficient effect on the reduction of pain	Subchondral bone marrow concentrate can postpone or avoid TKA	Hernigou et al.^[^ ^167^ ^]^ (N/T)
Osteoporosis (all patients had previous fragility fractures)	Non‐matched cord blood mononuclear cells (allogeneic)	6 × 10^7^ cells dose^−1^, at 4‐week intervals, 4 times in total	8	3	subcutaneously injected	Increase in insulin‐like growth factor 1 and BMD levels	Treatment increases the level of insulin‐like growth factor 1 to increase BMD	Li et al.^[^ ^168^ ^]^ (N/T)
Degenerative disc disease and low back pain	BM‐MSCs (autologous)	252 ml marrow per patient was harvested from the bilateral iliac crest	41	34.5	Operative placement	An alternative way to promote bone repair and regeneration	After 34.5 months of treatment, 95% of cases had good spinal fusion results	Gan et al.^[^ ^169^ ^]^ (N/T)
Degenerative disc disease and low back pain (L4‐L5 or L5‐S1)	BM‐MSCs (autologous)	cells were harvested and the final product containing 0.5–1.5 × 10^6^ cells kg^−1^ from the patient in a sterile cell suspension	11	12	Operative implantation	No adverse events; improvement on the visual analog scale (VAS) and ODI scores	The use of autologous MSCs for spine fusion in patients with monosegmental DDD is feasible and safe	Blanco et al.^[^ ^170^ ^]^ (NCT 0 151 3694)
Degenerative disc disease and low back pain (nonsurgical options limited)	BM‐MSCs (autologous)	A BMC volume of 7 mL (6 mL for injection and 1 mL for cell analysis) was prepared for injection	26	12	Local injection	No adverse events; reduction in ODI and VAS	Mesenchymal cell concentration can effectively reduce pain	Pettine et al.^[^ ^171^ ^]^ (N/T)
Degenerative disc disease and low back pain (nonsurgical options limited)	MPCs (allogeneic)	25 × 10 cells per segment under local anesthesia (MSCs group)	100	36	intradiscal injection	Improvement in VAS and ODI; no severe adverse events	Allogeneic MSC therapy provides pain relief and significantly improves disc quality	Noriega et al.^[^ ^172^ ^]^ (NCT 0 129 0367)
Degenerative disc disease and low back pain (modified Pfirrmann score of 3–6)	BM‐MSCs (allogeneic)	6 million MPCs versus 18 million MPCs versus the control group	24	12	intradiscal injection	improvement in algo functional indices versus the controls	Intradiscal injection of MPCs could be a safe, effective, durable, and minimally invasive therapy	Amirdelfan et al.^[^ ^173^ ^]^ (NCT 0 186 0417)
Degenerative disc disease and low back pain (Pfirrmann's grade III disc degeneration)	BM‐MSCs (autologous) and NP cells	One million activated NP cells were transplanted into the degenerated disc adjacent to the fused level at 7 days after the first fusion surgery	9	36	Transplantation	No adverse effects; confirmation of the safety and minimal efficacy of the NP cells transplantation	Activated NP cell transplantation is safe and can slow the further degeneration of human intervertebral discs	Mochida et al.^[^ ^114^ ^]^ (N/A)
Sarcopenia	N/A	N/A	N/A	N/A	N/A	N/A	N/A	N/A

MSCs, mesenchymal stem cells; AD‐MSCs, adipose‐derived stem cells; BM‐MSCs, bone marrow‐derived mesenchymal stem cells; UC‐MSCs, umbilical cord‐derived mesenchymal stem cells; MPCs, mesenchymal precursor cells; IA injection, intra‐articular injection; WOMAC, Western Ontario and McMaster Universities Arthritis Index; VAS, visual analog scale; ODI, Oswestry Disability Index; N/A, not available.

**Table 2:** Clinical trials investigating cell‐based therapies for OA, osteoporosis, and DDDs

Indication (stage)	Cell type (source)	Dosage and usage	No. of Subjects	Duration [Months]	Delivery Methods	Clinical Measure	Trial ID
OA, Knee (N/A)	MSCs (autologous)	Twenty million cells were injected into the discs	260	17	IA injection	Change of WOMAC and VAS scores from baseline	(NCT 0 399 0805)
OA, Knee (N/A)	ESCs (allogenic)	injection of 1 × 1 ml of amniotic tissue allograft	88	33	IA injection	Knee joint chemical profile; VAS scale and function	(NCT 0 340 8145)
OA, Knee (N/A)	HUMCWJ (allogenic)	SURGENEX SurForce (1 cc) allograft placental‐based tissue matrix, will be injected using a 22 gauge, 2‐inch needle (intervention)	60	17	IA injection	Change in WOMAC and joint pain during activities	(NCT 0 333 7243)
OA, Knee (N/A)	ADSCs (allogeneic)	6.4 × 10^−7^ cells versus 3.2 × 10^−7^ cells versus 1.6 × 10^−7^ cells versus the control group	57	63	IA injection	Changes from baseline to post‐treatment of WOMAC; incidence of adverse events	(NCT 0 278 4964)
OA, Knee (N/A)	Human UC‐MSCs	N/A	50	24	IA injection	Changes in VAS score and Kellgren–Lawrence Score after injection	(NCT 0 516 0831)
OA, Knee (nonsurgical options limited)	ADPSCs (allogenic)	N/A	53	36	IA injection	Improvements in joint function, pain, quality of life, and cartilage regeneration	(NCT 0 216 2693)
OA, Knee (nonsurgical options limited)	AD‐MSCs and BM‐MSCs	BM (10 cc) versus AD (10 cc) versus both (5 cc) injection	54	15	IA injection	Evaluation of the knee pain and function by using VAS score and WOMAC index	(NCT 0 435 1932)
OA, Knee (mild to moderate symptoms)	MSC derived exosomes	Intra‐articular knee injection of exosomes (3–5 × 10^11^ particles) derived from allogeneic mesenchymal stromal cells. Single dose.	10	24	IA injection	Adverse event; Incidence of injection‐related pain according to VAS scale; pain reduction	(NCT 0 506 0107)
OA, Knee	iPSCs	N/A	N/A	N/A	N/A	N/A	N/A
Osteoporosis (N/A)	BMSCs (autologous)	Cells will be infused intravenously on Day 0. The first four patients will receive a single dose of 2 million cells kg^−1^ and the last six patients will receive a single dose of 5 million cells kg^−1^.	10	32	IV	Rate of serious and non‐serious adverse events related to the procedure	(NCT 0 256 6655)
Osteoporosis (nonsurgical options limited)	Pre‐osteoblastic cells	Each patient will undergo a single intravenous administration of PREOB	20	35	IV	Cell biodistribution after intravenous infusion; incidence of any adverse events	(NCT 0 206 1995)
Intervertebral disc degeneration	ADC (autologous)	NOVOCART Disc plus versus NOVOCART Disc basic versus control group; dosage: N/A	120	106	Surgical implant	ODI index at follow‐up visit; the MRI‐signal of the disc; pain evaluation	(NCT 0 164 0457)
Degenerative disc disease	MPCs	High dose versus low dose MPCs versus control group	100	48	Local injection	Determination of the overall safety of MPCs; incidence of any adverse events	(NCT 0 129 0367)
Degenerative disc disease (nonsurgical options limited)	Osteoblastic cells (allogenic)	Each patient will undergo a single administration of ALLOB/ceramic scaffold mix into the lumbar interbody fusion site under anesthesia	38	75	Local injection	Assessment of lumbar fusion progression and functional disability	(NCT 0 220 5138)
Degenerative disc Disease and low back pain (N/A)	PRP enriched with exosomes	Platelet‐rich plasma (PRP) with exosomes vs Normal saline; dosage: N/A	30	11	Local injection	VAS scale; RDQ questionnaire; SF 36 health questionnaire	(NCT 0 484 9429)
Degenerative disc disease (nonsurgical options limited))	Human UC‐MSCs	Twenty million human umbilical cord mesenchymal stem cells will be immediately injected into the degenerative discs of each patient	20	36	Intradiscal injection	Changes from baseline in Lumbar disc signaling values from magnetic resonance imaging; VAS scale; ODI index	(NCT 0 441 4592)
Sarcopenia	N/A	N/A	N/A	N/A	N/A	N/A	N/A

ESCs, embryonic stem cells; HUMCWJ, mesenchymal stem cells derived from human umbilical cord Wharton's Jelly; ADSCs, adipose‐derived stem cells; BM‐MSCs, bone marrow‐derived mesenchymal stem cells; ADPSCs, adipose tissue‐derived mesenchymal progenitor cells; ADCT, autologous disc chondrocyte transplantation; MPCs, mesenchymal precursor cells; VCBM, viable cell bone matrix; PRP, platelet rich plasma; RDQ, Roland morris disability; IA injection, intra‐articular injection; IV, intravenous infusion; WOMAC, Western Ontario and McMaster Universities Arthritis Index; N/A, not available.

Ongoing research and advancements in cell‐based therapies have provided new opportunities for the clinical treatment of OA. Current cell types and derivatives include MSCs, iPSCs, and MSC‐derived exosomes. The number of clinical trials on the use of MSCs in the treatment of OA is increasing, and goals have gradually shifted from safety evaluation to exploration of efficacy, such as pain improvement.^[^ ^160^, ^161^, ^162^, ^163^, ^164^, ^165^, ^166^, ^167^ ^]^ A clinical study testing the efficacy of allogeneic BM‐MSCs in 140 patients following staged bilateral total knee arthroplasty (TKA) showed that subchondral bone cell‐based therapy (compared to TKA) had a sufficient effect on pain relief and postponed or prevented the need for TKAs in the contralateral joints of patients with bilateral OA.^[^ ^167^ ^]^ Although BM‐MSCs are the most common source of MSCs, many studies on AD‐MSC‐based therapies have been published.^[^ ^160^, ^166^ ^]^ A randomized controlled trial analyzed the safety and efficacy of intra‐articular AD‐MSC injections in 30 patients with OA. Patients who received AD‐MSCs showed clinically significant improvements in pain and function. Magnetic resonance imaging of the knee indicated changes in disease progression.^[^ ^166^ ^]^

MSC‐derived exosomes may inhibit the progression of articular cartilage degeneration, and their efficacy in treating OA has been analyzed. Unfortunately, there is currently only one registered phase 1 clinical trial on the use of exosomes for the treatment of OA. Patients have not yet been recruited and the results are expected to be obtained by 2023. iPSCs obtained from somatic cell reprogramming can theoretically be used to create specific chondrocytes in patients with OA and are expected to be a new source of therapeutic stem cells. However, current clinical research on iPSCs is still in the preliminary stages, and relevant clinical trials have not yet been conducted. In addition to the ethical issues mentioned above, other factors limit the applications of ESCs and iPSCs. Both ESCs and iPSCs can form tumors, especially teratomas, which is the biggest obstacle in the development of stem cell‐based therapies. iPSCs are more likely to form tumors than ESCs.^[^ ^174^ ^]^ Currently, the characteristics of iPSCs are not fully understood, and pure populations of differentiated derivatives cannot be obtained during stem cell differentiation. Most importantly, the generation of iPSCs and the differentiation of the corresponding cell lineages from iPSCs are extremely inefficient, which seriously restricts their application.

Clinical trials on the safety and efficacy of using stem and IVD cells in the treatment of DDDs have been conducted.^[^ ^114^, ^170^, ^171^, ^172^, ^173^ ^]^ These studies have focused on improving pain and evaluating disabilities in patients receiving cell‐based therapies for DDDs. Studies have shown significant improvements in pain and motor function after the injection of BM‐MSCs.^[^ ^170^, ^171^, ^172^ ^]^ However, long‐term improvements have not been reported due to the short follow‐up period in currently published studies. In addition, animal experiments have shown that the coculture of NP cells and MSCs can upregulate the activity of NP cells and significantly delay IVD degeneration. In contrast, a recent clinical trial found no such benefits in humans.^[^ ^114^ ^]^

Most clinical studies on the treatment of osteoporosis using cell‐based therapies are currently underway (Table 2). In China, only one clinical trial has been conducted on the clinical efficacy of non‐matched allogeneic cord blood mononuclear cells in the treatment of osteoporosis.^[^ ^168^ ^]^ Eight patients with osteoporosis received intermittent cell‐based therapy for 3 months. The results showed an increase in the lumbar spine bone mineral density. No registered or reported trials have investigated the effects of cell‐based therapy on sarcopenia.

The safety and efficacy of cell‐based therapies for DMDs have been established. The growing number of clinical trials and encouraging results support this hypothesis. These findings are of profound significance for future scientific research and translational applications. Nevertheless, the research efficacy does not indicate clinical effectiveness and further studies are needed to determine whether cell‐based therapies can span carefully minimized clinical differences. Given the wide heterogeneity in disease selection, patient inclusion and exclusion criteria, and outcome indicators in different clinical studies, the overall efficacy of cell therapy requires further clarification. Therefore, future studies are needed to further improve the level of standardization and focus on the long‐term therapeutic effects of different cell therapy regimens.

## 6 Future Directions and Approaches to Improve the Efficiency of Cell‐Based Therapies

Despite ongoing progress toward developing effective cell‐based treatments for DMDs, creating clinically and commercially viable products requires overcoming a series of challenges. Donor‐cell variability, transplantation viability, modification efficiency, and scalability from preclinical to clinical use are among the most prominent issues. These challenges are encountered in multiple overlapping steps in the development of treatments from bench to bedside, including the identification of cell sources, surface or content modifications, manufacture, and delivery. Recently, several attempts have been made to overcome these challenges, including the use of gene manipulation technologies, 3D culture technologies, and biomaterials.

### 6.1 Gene Manipulation Technologies

Researchers have used gene manipulation technologies to efficiently modify target cells. The most commonly used technology is clustered regularly interspaced short palindromic repeat (CRISPR)‐associated protein 9 (Cas9). CRISPR‐Cas9 gene editing technology uses artificially designed single‐guide RNAs to target specific genome sequences and guide Cas9 proteases to these sites for efficient DNA cleavage. This cleavage forms a double‐stranded break, which, when repaired, results in gene knockout or knock‐in. CRISPR‐Cas9 is one of the most efficient, simple, low‐cost, and easy‐to‐use gene editing and modification technologies and has become the mainstream gene editing system used today.^[^ ^175^ ^]^ Other gene editing technologies include zinc finger nucleases, transcription activator‐like effector nucleases, and the next‐generation ARC nuclease platform ARCUS. Each of these technologies has its own advantages and disadvantages and all are being developed.

In cell therapy, gene manipulation technologies primarily improve efficiency by manipulating target cells. These technologies can be used to implement two types of changes. One is gain‐of‐function, which allows cells to acquire a specific function or exhibit a particular phenotype. By gaining function, cells can increase their adhesion, become more convenient for enrichment at the target site, and express specific functional proteins. For example, tumor‐specific receptors (e.g., T cell receptors) are inserted into T cells using CRISPR‐Cas9 to better recognize tumor antigens, thereby significantly improving the efficiency of tumor treatment.^[^ ^176^, ^177^, ^178^ ^]^

Another type of change is loss‐of‐function, which interferes with gene expression and reduces the expression of a particular protein. In cell therapy, loss‐of‐function is typically used to reduce cell immunogenicity. Gene editing tools can also be used to simultaneously achieve the above two functions. For example, Deuse et al. modified human iPSCs by editing the coding sequences of *β*2‐microglubulin and CIITA (the main regulatory site of MHC class II) in the genome and introduced the CD47 gene sequence through a lentivirus to obtain iPSCs that did not simultaneously express HLA class I/II and overexpressed CD47. Engineered iPSCs are significantly less immunogenic, resulting in reduced immune rejection in allogeneic cell‐based therapies.^[^ ^179^ ^]^

There are concerns about the use of cell‐based therapeutic products containing recombinant viruses as delivery vehicles. A dilemma exists as the transient transfection period is not long enough to achieve therapeutic goals, while permanently transfected integrated viral vectors insert foreign genes into the whole genome, potentially causing disastrous results, such as the occurrence of tumors. In addition, systems, such as CRISPR‐Cas9, need to be studied further to increase their efficiency and reduce off‐target effects.^[^ ^180^, ^181^ ^]^ Along with the rapid development of gene editing techniques, more attempts need to be made to achieve optimal therapeutic effects with the lowest possible level of safety risks.

### 6.2 3D Culture Technology

3D culture technology may be the key to enhancing the efficiency of cell therapies in the preclinical and clinical stages.^[^ ^182^ ^]^ In 3D cell culture systems, cells are in an in vitro environment that allows them to grow in all directions, including in vivo. 3D cell culture enables complex cell–cell and cell–ECM interactions so that cells can be cultured in a more physiologically relevant environment that is conducive to their biological functions.^[^ ^183^ ^]^ Compared to traditional 2D cell cultures, 3D cell culture systems have a more realistic microenvironment. The morphology of cells in the 3D culture system closely resembles their natural shape. For instance, MSCs have been found to exhibit a spindle form in monolayer culture but display a round morphology when cultivated under stirring conditions using an ultra‐low attachment plate and other 3D materials.^[^ ^184^ ^]^ In addition, there are more cell connections, which allow for more cell‐to‐cell communication. The expression of genes and proteins in 3D‐cultured cells is similar to that in cells in vivo. Fontoura et al. used synthetic scaffolds for 3D cultures of B16 F10 murine melanoma cells and 4T1 murine breast cancer cells. In comparison with 2D cultures, RNA expression data supported the commonalities between 3D and in vivo groups.^[^ ^185^ ^]^ In addition, these cells responded more quickly to external mechanical and chemical stimuli and displayed a more realistic drug response.^[^ ^186^ ^]^ In summary, the cells in 3D culture environments resemble those in vivo. Therefore, 3D cell culture systems provide a more realistic reference for in vitro experiments and are potential alternatives to time‐consuming and expensive animal models.

**Figure 3:**
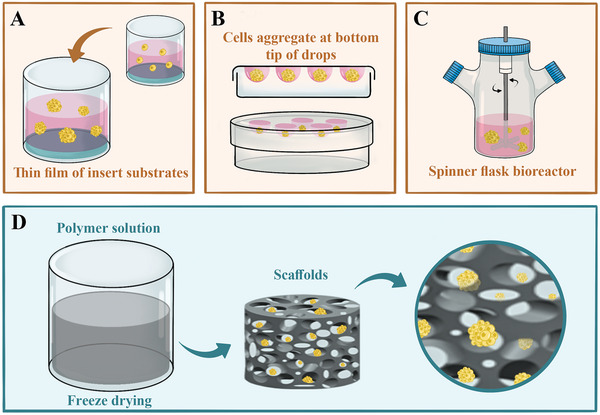
Types of 3D cell culture systems. A) Liquid overlay. B) Cells aggregate at the bottom tips of drops. C) Suspension bioreactor. D) Scaffold‐based technique.

Many 3D cell culture systems have been described in previous studies (**Figure** **3**). Generally, these can be classified into two categories: scaffold‐free and scaffold‐based. In addition, 3D cell culture systems can be made to be more efficient by integrating them with cutting‐edge technologies, such as microcarriers and microfluidic systems.^[^ ^187^, ^188^, ^189^ ^]^ Commonly used techniques are briefly described below.

### 6.3 Biomaterials

Various types of biomaterials have been used to improve the differentiation, delivery, retention, and safety of therapeutic cells. Materials ranging from degradable hydrogels to non‐degradable metals can be used to meet the treatment needs for different diseases and personalized cell therapies.

Biomaterials also provide additional signals that induce cell differentiation. During in vitro cell culture, stem cell differentiation requires soluble factors as well as physical and chemical stimuli. It has been suggested that the composition, topography, and mechanical properties of biomaterials play roles in regulating stem cell properties.^[^ ^190^ ^]^ Huebsch et al. used injectable void‐forming hydrogels to improve the osteogenic differentiation and deployment of MSCs in vitro. Researchers have demonstrated that the elasticity of hydrogels regulates bone regeneration, with optimal bone formation at 60 kPa.^[^ ^191^ ^]^ Wen et al. demonstrated that the matrix stiffness of polyacrylamide gels is a key factor in regulating the osteogenic and adipogenic differentiation of marrow‐derived MSCs.^[^ ^192^ ^]^ Ye et al. proposed that the functional binding motifs in biomaterials influence the fate of stem cells. They bound cell adhesive arginine‐glycine‐aspartate peptides to poly(ethylene glycol) hydrogel surfaces and found that this modification affected the differentiation of rat MSCs.^[^ ^193^ ^]^

Biomaterials also provide structural support when used as scaffolds to improve cell delivery and facilitate cell retention during therapy. When injected or administered locally, biomaterials protect cells from mechanical forces and provide structural support, enhancing cellular retention and therapeutic effectiveness. Wang et al. designed a novel injectable elastin‐like protein and hyaluronic acid hydrogel with improved structural integrity. This hydrogel provides mechanical protection to encapsulated MSCs during syringe needle injections, enables cell culture for 3 weeks after injection, and preserves the ability of cells to differentiate into multiple lineages.^[^ ^194^ ^]^ In addition, biomaterials can carry therapeutic agents, such as growth factors and immunomodulatory molecules, thereby coordinating with therapeutic cells to endow synergistic effects. Prokoph et al. designed starPEG‐heparin hydrogels functionalized with SDF‐1*α*, which could guide stem cell migration toward the injured site. The gels significantly enhanced the migration of early endothelial progenitor cells toward the concentration gradients of hydrogel‐delivered SDF‐1*α* in vitro and improved vascularization in a mouse model.^[^ ^195^ ^]^

It is worth noting that scaffolds play a unique role in the treatment of degenerative diseases of the skeletal system. The microstructure and mechanical characteristics of the scaffold can be modified by adjusting the physical properties of the scaffold material and precisely guiding the directed differentiation of stem cells. Zhou et al. revealed that various fiber sizes efficiently promoted the differentiation of AF‐derived stem cells into certain cells corresponding to the types of cells in distinct AF zones, using poly L‐lactic acid fibrous scaffolds to culture AF.^[^ ^196^ ^]^ To recreate the angle‐ply architecture of AF, Liu et al. created high‐resolution PCL scaffolds that were later assembled into AF constructs. The tissue‐engineered in vivo IVD performance contributed to disc height maintenance, minimized NP water content loss, and partially restored IVD biomechanical function.^[^ ^197^ ^]^ These studies laid a strong foundation for the management of bone degenerative diseases and highlighted potential new treatment strategies.

## 7 Conclusions and Outlook

The clinical application of cell‐based therapies has entered a significant stage of research in regenerative medicine. The value of the global cell therapy market is expected to reach US $21.06 billion from 2022 to 2026 and its growth will accelerate at a compound annual growth rate of 56.79%. Excellent progress has been made in the treatment of Parkinson's disease, tumors, and cardiovascular diseases. Cell‐based therapy is the most promising strategy for the treatment of degenerative disorders. Given their differentiation potential, immunomodulatory capabilities, and paracrine activity, stem cells have made it feasible to overcome the limitations of traditional therapies, which can only slow disease progression. With the help of numerous cutting‐edge technologies, including 3D culture, biomaterials, and gene editing technologies, cell therapy has become more effective and precise and has a wide range of potential applications.

However, various problems with cell‐based therapies still need to be resolved. Although the directional differentiation and paracrine abilities of stem cells have been discovered, the underlying molecular mechanisms of these functions have not yet been clarified, and other mechanisms of action of stem cells are unclear. The cellular‐ and molecular‐level pathological changes in specific degenerative diseases require further investigation. The lack of understanding of these changes limits the elucidation of mechanisms underlying cell‐based therapies. In addition, there is an urgent need to establish standardized processes. Currently, cell‐based therapy is still in the exploratory stage, and the strategies used differ between studies. The sources of cells used to treat different diseases are also inconsistent. The methods of their acquisition are different, whereas the methods of their isolation, expansion, and culture are distinct. In addition, there is no standardized approach for the dose, frequency, or administration of cells, such as local or systemic. These inconsistencies lead to an inability to compare and evaluate different approaches, limiting the development of cell‐based therapies.

However, the safety of these cell‐based therapies has not been fully verified. The first issue to consider is tumorigenicity. Tumor formation can be induced in multiple steps, including cell acquisition, isolation, culturing, genome editing, expansion, and implantation. Incorrect gene patterning, the induction of various reprogramming factors, and genetic abnormalities are important causes of tumorigenicity. The formation of teratomas is the most serious problem in the transplantation of iPSCs and ESCs and may be caused by the abnormal growth of stem cells of a specific lineage in vivo. Additionally, the introduction of tumorigenic genes, including c‐Myc, into iPSCs is an important cause of tumor induction. Cells must be expanded in vitro before transplantation, which inevitably affects the stability of genetic information. Abnormalities in chromosomes with larger mutations can be monitored by karyotyping; however, small genetic alterations, such as single‐nucleotide variants and copy number variations, are more difficult to assess. Next‐generation sequencing technology may be an option for detecting genetic alterations.^[^ ^11^ ^]^

Immune rejection is another important problem that must be solved before the clinical application of cell‐based therapies. However, allogeneic cell transplantation can induce immune rejection in the host, resulting in serious adverse clinical consequences. Autologous transplantation of iPSCs harvested from patient cells is one of the most promising cell‐based therapeutic options because it theoretically avoids immune rejection. Nonetheless, studies have suggested that abnormal gene expression and mitochondrial mutations promote the immunogenicity of autologous iPSCs. Exosomes are considered to be less immunogenic and are becoming increasingly important in cell‐based therapies. Basic research on the exosome‐related mechanisms involved in the maintenance of tissue homeostasis within the musculoskeletal system will facilitate the selection of appropriate cell sources for exosome production. Increasing the biocompatibility of exosomes could augment their efficacy in treating DMDs. However, the efficacy of cell‐based therapies has not yet been validated in long‐term follow‐up studies. Therefore, long‐term randomized controlled trials are required to clarify the efficacy and safety of cell‐based therapies.

## Conflict of Interest

The authors declare no conflict of interest.
